# Trauma-Informed Approaches in the Context of Cancer Care in Canada and the United States: A Scoping Review

**DOI:** 10.1177/15248380221120836

**Published:** 2022-09-09

**Authors:** Cara A. Davidson, Kelly Kennedy, Kimberley T. Jackson

**Affiliations:** 1 The University of Western Ontario, London, Canada

**Keywords:** scoping review, cancer, oncology, trauma-informed approaches, trauma-informed care, Canada, United States

## Abstract

Cancer is predominantly understood as a physical condition, but the experience of cancer is often psychologically challenging and has potential to be traumatic. Some people also experience re-traumatization during cancer because of previous, non-cancer-related trauma, such as intimate partner violence or adverse childhood experiences. A trauma-informed approach to care (TIC) has potential to enhance care and outcomes; however, literature regarding cancer-related TIC is limited. Accordingly, the objective of this scoping review was to identify what is known from existing literature about trauma-informed approaches to cancer care in Canada and the United States. A scoping review (using Arksey and O’Malley’s (2005) framework) was conducted. The PsycINFO, CINAHL, MEDLINE (Ovid), Embase (Ovid), and Scopus databases, key journals, organizations, and reference lists were searched in February 2022. In total, 124 sources met the review criteria and 13 were included in the final review. Analysis included a basic descriptive summary and deductive thematic analysis using conceptual categories. Theorizations, applications, effectiveness, and feasibility of TIC were compiled, and gaps in TIC and recommendations for TIC were identified. TIC appeared to be growing in popularity and promising for improving cancer outcomes; however, gaps in the theorization, effectiveness, and feasibility of TIC persisted. Many recommendations for the application of TIC were not issued based on a strong body of evidence due to a lack of available literature. Further research is required to develop evidence-based recommendations regarding TIC related to cancer. A systematic review and meta-analysis would be warranted upon literature proliferation.

In 2020 alone, an estimated 2.5 million people in Canada and the United States were diagnosed with cancer (Globocan, 2020). While cancer is predominantly understood as a physical condition, the experience of cancer is often psychologically challenging due to previous traumas that intersect to exacerbate cancer-related challenges (Cordova et al., 2017).

Trauma is a complex inner experience, understood as the experience of, and response to, an overwhelmingly negative event or series of events (Ponic et al., 2021). The effects of trauma can extend throughout the lifespan, even years after the traumatic event has occurred (Ponic et al., 2021). Common examples of traumatic events include major health events such as cancer, adverse childhood experiences, gender-based violence, natural disasters, and financial stressors (Canadian Mental Health Association, 2021). However, as noted by the Substance Abuse and Mental Health Services Administration (SAMHSA, 2014a), “It is not just the event itself that determines whether something is traumatic, but also the individual’s experience of the event” (pp. 17).

## Cancer Risk, Diagnosis, Treatment, and Survivorship

Trauma and cancer are often presumed to intersect at an individual’s cancer diagnosis, as cancer diagnosis itself is considered a potentially traumatic event (Cordova et al., 2017; Marziliano et al., 2020). However, the intersection of trauma with cancer can occur prior to diagnosis—during cancer-risk behaviors and cancer screening activities—and extend beyond diagnosis into treatment, remission, and survivorship care. Re-traumatization, or the perceived potential for re-traumatization, can compromise cancer outcomes by jeopardizing patients’ participation in cancer screening, treatment, and follow-up care (Cordova et al., 2017; Hanna et al., 2020; Regal et al., 2020).

Strong links have been identified between the experience of trauma and certain behaviors that may lead to cancer (Ports et al., 2019). For example, substance use, poor diet, and high-risk sexual behaviors—often employed as coping mechanisms for trauma—are linked to an increased risk of cancer. For example, a 2019 review by Ports et al. indicated that adults who had experienced childhood adversity were more likely to consume nicotine, alcohol, and/or excessive sugar to cope, thus increasing their risk of cancer. In addition, Senn et al. (2008) and Stubbs and Szoeke (2021) reported that adults who had experienced childhood sexual abuse, and women who experienced intimate partner violence were more likely to engage in high-risk sexual behaviors, which increased their risk of exposure to sexually transmissible infections and risk of cervical cancer.

While cancer screening and diagnostics are important aspects of cancer prevention and/or care, these procedures can be re-triggering for patients with trauma histories. In addition, Cordova et al. (2017) identified diagnostic testing and stressful waiting periods as potentially traumatic events in the cancer screening period. Moreover, Kohler et al. (2021) described that some women with histories of sexual trauma were re-traumatized during Pap screenings for cervical cancer, while others chose to avoid Pap screening entirely to avoid re-traumatization. Studies by Farley et al. (2001, 2002) also described that women with trauma histories may avoid seeking mammograms as preventive breast cancer care to avoid re-traumatization. Overall, a lack of interaction with timely cancer screening practices can compromise future health outcomes because of late detection of malignancies and delays in the delivery of quality cancer care (Primeau et al., 2014; Ramachandran et al., 2015; Vose, 2019).

Receiving a diagnosis of cancer requires psychological adjustments and can be perceived as a traumatic experience (Andrykowski & Kangas, 2010; Cordova et al., 2017). As noted by Lawson and Lawson (2018), some cancer patients view their cancer diagnosis through the lens of previous trauma; patients with trauma history have been found to be significantly more likely to experience fatigue, depression, or stress symptoms and elevated levels of inflammatory markers. Understanding cancer diagnosis as traumatic is well-established in the context of breast cancer (Glanz & Lerman, 1992; Mehnert & Koch, 2007), and is beginning to be understood across other forms of cancer as well (Gieseler et al., 2018).

Prior traumatic experiences can influence or shape decision-making around cancer treatment (Cordova et al., 2017). For example, qualitative work by Dionigi et al. (2019) described the experience of a 48-year-old woman who navigated metastatic breast cancer in the context of intimate partner violence. In this case, the woman chose to forgo breast reconstruction after mastectomy to make the signs of cancer “as evident as possible” (p. 3, para. 3; Dionigi et al., 2019), using her sick body as a shield to protect herself from her abusive partner. It has also been noted that patients with a history of adverse childhood experiences had reduced treatment adherence and greater relational difficulties with providers (Regal et al., 2020). Trauma can also arise from negative healthcare experiences (Hall & Hall, 2013), which in turn may influence patients’ decision-making regarding treatment (SAMHSA, 2014b). Common sources of healthcare-related trauma include discrimination and stigma (i.e., racism, sexism, ableism, etc.; Stangl et al., 2019). Healthcare-related trauma can occur at any point in a patient’s care journey, including prior to or during cancer treatment and during non cancer-related care. Regardless of the source, experiences of healthcare-related trauma can contribute to cancer patients choosing to delay and/or avoid care (Quach et al., 2012). Given the importance of early identification and treatment to mitigate cancer-related morbidity and mortality, treatment non-adherence is a significant concern. To highlight, a systematic review and meta-analysis by Hanna et al. (2020) reported that even a four-week delay in cancer treatment is associated with increased mortality. As such, the interaction of trauma with cancer treatment can exacerbate cancer-related morbidity and mortality.

More positively, with improvements in technology and healthcare, larger proportions of people with cancer are surviving (Siegel et al., 2020). However, even after successful cancer treatment, survivorship typically includes years of routine surveillance care, including follow-up scans and oncology appointments, which can also be re-traumatizing for patients (Cordova & Andrykowski, 2003; Ghazali et al., 2013). Moreover, the re-appearance of cancer symptoms (Ghazali et al., 2013; Moye et al., 2010), or recurrence of cancer itself (Cella et al., 1990), are also known to be potentially traumatic. Even the death of a public figure due to cancer can be traumatic for patients in remission (Ghazali et al., 2013). Experiencing trauma responses beyond initial cancer treatment can result in delayed and/or missed appointments, which is known to compromise health outcomes (Cordova et al., 2017).

## Trauma-Informed Approaches to Care

It is important to note that trauma responses to cancer are considered normative, not pathological, given the threat to life and well-being (Kazak & Noll, 2015). However, trauma becomes problematic when it negatively affects cancer outcomes. A promising response to improving outcomes for cancer patients affected by trauma is a trauma-informed approach. The SAMHSA (2014a) has defined a trauma-informed approach as a program, organization, or system that

“Realizes the widespread impact of trauma and understands potential paths for recovery; recognizes the signs and symptoms of trauma in clients, families, staff, and others involved with the system; and responds by fully integrating knowledge about trauma into policies, procedures, and practices, and seeks to actively resist re-traumatization.” (p. 9)

A trauma-informed approach to care (TIC) is distinct from trauma-sensitive, trauma-focused, and trauma-based forms of care, because it does not aim to elicit a description of the trauma, nor address it directly. Instead, TIC considers trauma as an essential consideration to prioritize optimal care of a health issue. A trauma-informed approach promotes patient healing and recovery through six principles (SAMHSA, 2014a): (1) safety, (2) trustworthiness and transparency, (3) peer support, (4) collaboration and mutuality, (5) empowerment, voice, and choice, and (6) cultural, historical, and gender issues. Wathen et al. (2021) have extended TIC to include violence, by adopting the term *trauma-and violence-informed care* (TVIC). TVIC expands TIC by accounting for the “intersecting impacts of systemic and interpersonal violence and structural inequities on a person’s life, emphasizing both historical and ongoing violence and their traumatic impacts.” (Wathen & Varcoe, 2021, p. 1). TIC has successfully improved mental health outcomes in multiple populations, including caregivers (Dugan et al., 2020), pregnant and postpartum women (Kim et al., 2021), mental health patients (Gatz et al., 2007), and acute care patients (Champagne & Stromberg, 2004). Given the important intersections of trauma and cancer care, it is reasonable that TIC also holds promise as an approach for cancer patients.

As noted by Kimberg and Wheeler (2019), due to its pervasive nature, “all healthcare providers will care for patients with histories of trauma” (p. 26, para. 1). Adding to its complexity, cancer-related trauma may also be experienced beyond the patients themselves, extending to their loved ones (e.g., parents and partners), requiring a family-focused care response (Cordova et al., 2017; Edwards & Clarke, 2004; Norberg et al., 2005). Recognizing this, recommendations have been issued to work toward a trauma-informed approach for patients (and families) with cancer to help providers prevent re-traumatization and improve health outcomes. However, research regarding TIC conceptualization and applications specific to cancer is limited. This makes it difficult for cancer care providers to understand the value of TIC across cancer populations, cancer types and stages, and medical providers.

Accordingly, the objective of this scoping review was to identify what is known from existing literature about trauma-informed approaches to cancer in Canada and the United States. This objective was ideally suited to a scoping review approach, as it serves to map the existing literature, identify conceptualizations, applications, and the efficacy of TIC, as well as determine gaps to inform future practice, policy, and research.

## Methods

A scoping review was conducted to map the literature as it pertains to trauma-informed care in the context of cancer. The review was informed by Arksey and O’Malley’s (2005) scoping review framework, inclusive of the enhancements proposed by Levac et al. (2010) and Peters et al. (2015). The five stages of the Arksey and O’Malley (2005) scoping review process were adhered to, including: (1) identifying the research question, (2) identifying relevant studies, (3) study selection, (4) charting the data, and (5) collating, summarizing, and reporting the results.

### Stage One: Identifying the Research Question

The research question developed for this review was: What is known from existing literature about trauma-informed approaches to cancer in Canada and the United States? A “wide” approach was employed such that the definition of TIC and the population receiving TIC could differ between articles (Arksey & O’Malley, 2005). For example, articles providing TIC to cancer patients and articles providing TIC to the families of cancer patients would each be relevant to this question. The research question was restricted to Canada and the United States to ensure a manageable number of studies for an exploratory scoping review and to ensure the population was applicable to knowledge users.

### Stage Two: Identifying Relevant Studies

Next, articles were compiled through searching databases, hand-searching key journals and relevant organizations, and checking reference lists (Arksey & O’Malley, 2005). Only English texts and sources based in Canada, or the United States were considered for inclusion. Only English texts were considered due to the language preferences and resource restrictions of the authors. No publication date limitations were imposed. Consultation with the research team’s institutional academic librarian was also conducted to develop, execute, and refine the search strategy.

#### Databases

The PsycINFO, CINAHL, MEDLINE (Ovid), Embase (Ovid), and Scopus databases were searched in February 2022 to compile literature from the following search terms: trauma-informed, violence-informed, trauma-and-violence-informed, cancer, and oncolog*. In accordance with Levac et al.’s (2010) and Peters et al.’s (2015) recommendations, the search strategy was piloted and continuously refined. Notably, potential suffixes to “trauma-informed” were initially included, but ultimately omitted to account for various suffixes in literature that described a trauma-informed approach (e.g., care, practice, precautions, medicine, and treatment). Results were limited to those in Canada and the United States and available in English, which returned 121 articles.

#### Hand-Searching Key Journals and Relevant Organizations

The Clarivate Incites tool was used to compile the top 10 journals (determined by 5-year impact factor as of 2022) across six relevant areas of literature: health care sciences and services, integrative and complementary medicine, multidisciplinary sciences, nursing, psychology, and oncology. It is known that electronic databases are not always complete, nor up to date, and that their abstracting services vary in detail (Arksey & O’Malley, 2005). These high-quality, popular journals were selected to ensure any relevant, new articles would not be overlooked. This method identified 34 distinct journals, all of which were searched. The websites of thirteen prominent organizations, Canadian Cancer Society (2022), American Cancer Society (2022), American Society for Clinical Oncology (2022), EQUIP Health Care (2020), World Health Organization (2022), Global Cancer Observatory (2022), International Agency for Research on Cancer (2022), Union for International Cancer Control (2022), World Cancer Congress (2022), International Cancer Control Partnership (2022), All.Can (2022), Movement for Global Mental Health (2022), and International Mental Health Association (2021) related to mental health, cancer, and trauma were also searched for scholarly materials. The search was limited to thirteen of the most popular cancer and health-related organizations for feasibility purposes, as this form of searching generates large numbers of references (Arksey & O’Malley, 2005). Two sources of potentially relevant literature and one relevant article that was a duplicate were identified through this method.

#### Reference Lists

The reference lists of the 12 relevant articles found through database searching were searched in full for additionally relevant articles. This identified one new article (Kazak et al., 2006).

### Stage Three: Study Selection

Following Levac et al.’s (2010) recommendation, two reviewers met to discuss inclusion criteria before independently reviewing abstracts and subsequent full texts. It was agreed that to be considered eligible for inclusion, studies had to report the use of trauma-informed care in the context of cancer, be conducted in Canada and/or the United States, be available in English text, and have an accessible full-text (i.e., no conference abstracts). This resulted in 13 articles included in the final review.

### Stage Four: Charting the Data

An iterative approach to data charting was employed as per Levac et al.’s (2010) and Peters et al.’s (2015) recommendation. The reviewers independently extracted data for the first five included studies and refined the extracted conceptual headings to better align with the objective prior to completing extraction (Levac et al., 2010; Peters et al., 2015). The final conceptual headings employed were: title, date, author(s), country, objective(s), key outcomes, design, analysis, type, method, context, and outcomes of TIC, population/sample, type and timing of cancer, inclusion/exclusion criteria, relevant results, and recommendations. Although efforts were made to complete the charting process for each article in full, variations in reporting by authors resulted in some incomplete data charting for some sources.

### Stage Five: Collating, Summarizing, and Reporting the Results

Articles were analyzed using basic descriptive summary analysis and deductive thematic analysis using conceptual categories (Levac et al., 2010; Peters et al., 2015). This involved developing an a priori template (Crabtree, 1999) to organize the analysis, which was based on the research question being asked. Analysis consisted of actively seeking out data to complete the following five conceptual categories: (1) theorizations of TIC, (2) applications of TIC, (3) effectiveness and feasibility of TIC, (4) gaps in TIC, and as recommended by Levac et al. (2010), and (5) recommendations for future policy, practice, and research.

## Results

**Figure 1.**
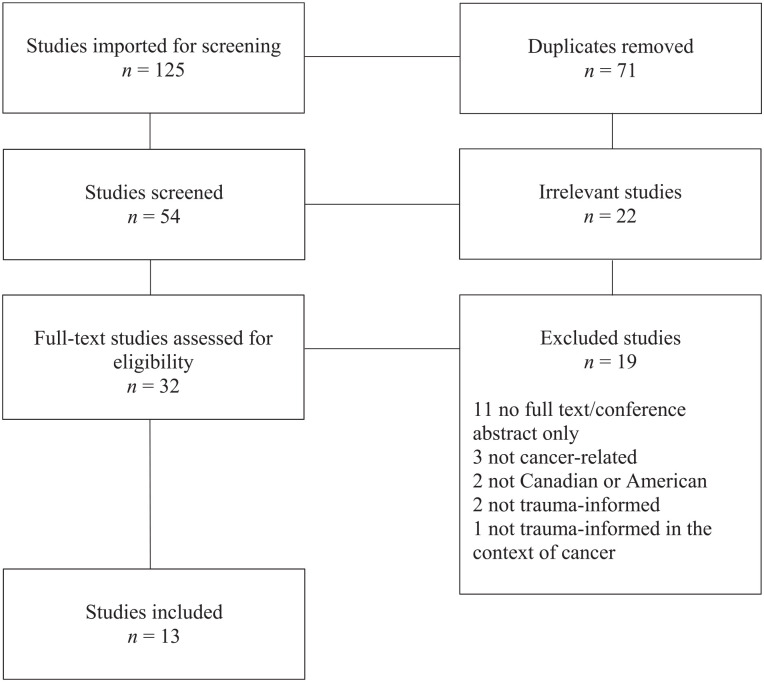
PRISMA diagram.

This scoping review aimed to identify what is known from the current literature about trauma-informed approaches in the context of cancer in Canada and the United States. Ultimately, 13 articles met this objective and included six various cancer types in both pediatric (Garcia et al., 2017; Kazak et al., 2005, 2006) and adult (Andrejko & Katrichis, 2022; Currie et al., 2019; Dhawan & LeBlanc, 2022; Kohler et al., 2021; Lawson & Lawson, 2018; Niebauer et al., 2021; Owens et al., 2021; Regal et al., 2020; Sayani et al., 2021; Suarez et al., 2021) patients (see Table 1 for a summary and Figure 1 for the PRISMA diagram). Seven studies included primary data regarding TIC (Andrejko & Katrichis, 2022; Kazak et al., 2005; Kohler et al., 2021; Niebauer et al., 2021; Owens et al., 2020; Regal et al., 2020; Sayani et al., 2021), one used secondary data (Suarez et al., 2021), and five used neither primary nor secondary forms of data (Currie et al., 2019; Dhawan & LeBlanc, 2022; Garcia et al., 2017; Kazak et al., 2006; Lawson & Lawson, 2018). A summary of critical findings is available in Supplemental Appendix A.

**Table 1.** Characteristics of Included Articles.

First Author Surname	Year	Design/Methods	Country	Primary Objective	Sample	Type of Cancer Experience	Type of Cancer
Andrejko	2022	Case study	United States	To demonstrate how a trauma-informed approach can be used to develop supportive interventions.	23-year-old female with cancer	Active malignancy	Acute myeloid leukemia
Currie	2019	Rationale and design for randomized trial	Canada	To evaluate the effect of trauma-informed group yoga, drumming, and psychoeducation compared to one another and control on tobacco use, alcohol use, and sugar-sweetened beverage consumption among community-based adults.	N/A	Cancer-risk behaviors (pre-cancer)	Not specified
Dhawan	2022	Commentary	United States	None stated.	N/A	Active malignancy	Hematological
Garcia	2017	Fictional case illustration	United States	None stated.	14-year-old male with cancer.	Active malignancy	Pediatric acute lymphocytic leukemia
Kazak	2005	Self-report questionnaire	United States	To report post-traumatic stress symptoms in mothers and fathers of children in current treatment for a pediatric malignancy.	Mothers (*n* = 119) and fathers (*n* = 52) from 125 families of children diagnosed with a pediatric malignancy.	Active malignancy	Pediatric leukemia, lymphoma, solid tumors, and brain tumors
Kazak	2006	Integrative model presentation	United States	To guide assessment and intervention for patients and families, a model for assessing and treating pediatric medical traumatic stress is presented that integrates the literature across pediatric conditions.	N/A	Active malignancy	Pediatric not specified
Kohler	2021	In-depth interviews	United States	To explore how trauma, especially sexual trauma, affects cervical cancer screening experiences, behaviors, and provider practices in the context of homelessness.	18 female patients (no history of cervical cancer) and 11 providers (primary care, gynecology, and internal medicine).	Routine cancer screening	Cervical
Lawson	2018	Commentary	United States	None stated.	N/A	Active malignancy	Not specified
Niebauer	2021	Exploratory qualitative design including an online survey and phone structured interview	United States	To evaluate breast cancer survivors’ perception of the relation between breast cancer development and both childhood trauma and stressful life events in adulthood.	50 women with a history of, or currently living with breast cancer.	Previous or current malignancy	Breast cancer
Owens	2020	Qualitative semi-structured interviews, design and implementation of pilot program, program evaluation surveys, and chart audits of electronic health data	United States	To implement and evaluate patient-centered reproductive healthcare services at a Seattle needle syringe program.	15 female clients of community organization serving women who inject drugs and 13 staff	Routine cancer screening	Cervical and ovarian
Regal	2020	Case example	United States	To utilize a case example to illustrate the potential utility of trauma-informed care within integrated care settings.	54-year-old Hispanic female patient with cancer	Active malignancy	Breast cancer
Sayani	2021	Semi-structured interviews	Canada	To understand the perspectives of physicians on access to lung cancer screening for individuals living with low income.	11 family physicians in primary care settings (3 male, 8 females).	Routine cancer screening	Lung cancer
Suarez	2021	Secondary statistical analysis of clinical trial data	Canada	To comprehensively assess the prevalence of adverse childhood experiences and association with health outcomes among transmasculine individuals.	150 transmasculine individuals	Routine cancer screening	Cervical cancer

### Theorizations of TIC

In total, five articles included a definition of TIC (Andrejko & Katrichis, 2022; Dhawan & LeBlanc, 2022; Lawson & Lawson, 2018; Regal et al., 2020; Sayani et al., 2021), while eight did not (Currie et al., 2019; Garcia et al., 2017; Kazak et al., 2005, 2006; Kohler et al., 2021; Niebauer et al., 2021; Owens et al., 2020; Suarez et al., 2021). The authors that did not operationalize TIC, employed it as a community intervention (Currie et al., 2019), medical intervention (Garcia et al., 2017; Kazak et al. 2005, 2006; Niebauer et al. 2021; Owens et al., 2020; Suarez et al., 2021), form of psychoeducation (Currie et al., 2019; Garcia et al., 2017), a health/cancer screening method (Garcia et al., 2017; Kohler et al., 2021), and/or a trauma screening method (Kohler et al., 2021; Suarez et al., 2021). Of the articles that operationalized the term, three utilized the definition of a trauma-informed approach as developed by the SAMHSA (2014a) which includes four assumptions and six main principles; Andrejko and Katrichis (2022) described all assumptions and principles of the SAMHSA (2014a) definition, but Dhawan and LeBlanc (2022) and Lawson and Lawson (2018) exclusively described the principles. According to SAMHSA (2014a), healthcare providers can utilize a trauma-informed framework to build trusting relationships with patients and improve patient-provider communication. The four assumptions of a trauma-informed approach to care include:

(1) realize the widespread impact of trauma and understand [the] potential paths for recovery;
(2) recognize the signs and symptoms of trauma in clients, families, staff, and others involved with the systems;
(3) respond by fully integrating knowledge about trauma into practice, procedures, and practices;
(4) resist re-traumatization. (SAMHSA, 2014a, p. 9)

Although Regal et al. (2020) and Sayani et al. (2021) did not adopt SAMHSA’s (2014a) definition of TIC, their conceptualizations aligned with the broader literature. Regal et al. (2020) adopted a definition from an article by Oral et al. (2016), describing TIC as a tertiary effort to prevent mental, social, and physical health consequences across the life span. However, Oral et al. (2016) built this definition based on the SAMHSA framework. Resultantly, Regal et al.’s conceptualization aligned with SAMHSA (2014a) by describing resisting re-traumatization, empowerment, transparency, and choice as central to TIC. Moreover, Sayani et al.’s (2021) conceptualization was loosely tied to SAMHSA’s sixth principle, which specifies cultural, historical, and gendered issues, by describing TIC as equity-oriented care that acknowledges and addresses the health impacts of structural violence. Resultantly, Sayani et al.’s (2021) definition of TIC aligned, albeit narrowly, with the broader literature. Overall, the SAMHSA (2014a) description of TIC appeared to be the dominant theorization of TIC in the context of cancer.

### Applications of TIC

How TIC was applied varied among the articles, with particular variation between screening and care delivery practices. One study in this review advocated for universal trauma screening (Regal et al., 2020), and two recommended targeted trauma screening (Kohler et al., 2021; Suarez et al., 2021). Two sources advocated for responsive TIC (i.e., TIC in response to a disclosure of trauma; Andrejko & Katrichis, 2022; Dhawan & LeBlanc, 2022) and two others recommended universal TIC provision (Kazak et al., 2006; Suarez et al., 2021). No sources of evidence advised against TIC. Applications of TIC were described in both community (Currie et al., 2019; Kazak et al., 2006) and healthcare (Andrejko & Katrichis, 2022; Garcia et al., 2017; Owens et al., 2020; Regal et al., 2020; Sayani et al., 2021) settings.

#### Community-based TIC

Four studies proposed TIC interventional efforts aimed at community-based adults (Currie et al., 2019) and pediatric oncology patients and their families (Garcia et al., 2017; Kazak et al., 2005, 2006). For the purposes of this review, “community” was understood as involving people at-risk of cancer, and/or any parties beyond the person with cancer. Currie et al. (2019) developed a proposal for evaluating the effect of trauma-informed group yoga, drumming, and psychoeducation on mood, immune system response, and trauma-related symptoms for adults at-risk of cancer. The authors proposed the use of twelve sessions, delivered weekly by trained professionals for each intervention. The goals of the group sessions were to teach individuals who have experienced trauma ways to connect the mind and body, to respond to and positively cope with stress. In pediatric applications, Kazak et al. (2005) prompted providers to employ toolkits to address familial needs regarding traumatic stress; the use of a three-phase pediatric medical traumatic stress (PMTS) model aimed at preventing traumatic stress through family-centered TIC was proposed. The model highlighted the importance of utilizing a multidisciplinary approach to develop interventions targeted at delivering trauma-informed treatment based on the patient’s subjective experience of medical events (Kazak et al., 2006). Similarly, Garcia et al. (2017) described the FOCUS theoretical model, an integrated approach to behavioral health screening and consultation, for pediatric oncology families. The model was composed of eight 60–90-minute sessions that emphasized traumatic stress reduction and resilience enhancement through trauma-informed screening procedures and family-resilience enhancement strategies (Garcia et al., 2017). Evidence suggests that trauma-informed family-centered interventions provides an opportunity for preventing traumatic stress (Kazak et al., 2006), increasing family resilience, and optimizing well-being (Garcia et al., 2017).

#### Healthcare settings

The remaining studies integrated, or proposed the integration of, TIC into care for people with cancer (Andrejko & Katrichis, 2022; Dhawan & LeBlanc, 2022; Garcia et al., 2017; Kohler et al., 2021; Lawson & Lawson, 2018; Niebauer et al., 2021; Owens et al., 2020; Regal et al., 2020; Sayani et al., 2021; Suarez et al., 2021). When describing the application of TIC in a healthcare setting, five studies focused on the importance of an interprofessional care team (Andrejko & Katrichis, 2022; Garcia et al., 2017; Kazak et al., 2006; Regal et al., 2020; Sayani et al., 2021). Andrejko and Katrichis (2022) described the involvement of physicians, social workers, case managers, pharmacists, registered dietitians, advanced practice providers, and clinical nurses during TIC. Garcia et al. (2017) identified that an interprofessional care team should include oncologists, social workers, nurses, patient navigators, child life specialists, and *embedded psychologists* whose involvement stemmed from the FOCUS intervention provider’s prompting. Garcia et al. (2017) also extended the care team to include a *curandero* (a traditional healer in Latin cultures) to provide culturally sensitive family-centered care. Regal et al. (2020) identified the interdisciplinary care team to include physicians, nurse practitioners, nurse navigators, counselors, and physical therapists. Sayani et al. (2021) identified nurses, social workers, and patient navigators as essential to an interprofessional care team. Kazak et al. (2006) did not specify providers, although they described TIC as including both medical and nursing practice. While the professional composition of care teams varied, it was evident that the interdisciplinary nature was important to the delivery of TIC.

Specific applications of TIC in care settings were not well-reported in the literature, with the exception of Dhawan and LeBlanc (2022) and Kohler et al. (2021), as the ways in which providers integrated TIC into their practice were often difficult to identify. Dhawan and LeBlanc (2022) provided an applications table that identified how each component of TIC could be applied to hematologic care, inclusive of potential action items for a hematology team. For example, within the safety component, a suggested application was “Be mindful that maladaptive behavior (e.g., smoking, high-risk behaviors, overeating, etc.) can be manifestations of emotional crises.” (p. 2), with the potential action item of “Screen for emotional distress at every visit.” (p. 2). However, screening tools were not suggested to facilitate identifying emotional distress. Similarly, Kohler et al. (2021) provided strategies for TIC in the context of pre-, during, and post-screening for cervical cancer. Many strategies had clear applications, such as, “Offer choice of provider gender to conduct Pap” during screening; however, others were unclear in how they may be applied, for example, to “Promote a sense of safety, trust, and dignity” in pre-screening (Kohler et al., 2021, Table 4). Other studies in the review were also missing detailed descriptions of the applications of TIC. For example, Lawson and Lawson (2018) noted that multiple opportunities were available to implement TIC into practice to mitigate trauma symptoms and distress but did not provide examples of such opportunities. Kohler et al. (2021) recommended creating a safe environment by gradually earning patient trust. This was achieved by delaying discussions of screening for trauma until patient-provider trust was established, although the mechanism to discern adequate trust was unclear. Additionally, Owens et al. (2020) reported the use of an obstetrician-gynecologist to train a female Advanced Registered Nurse Practitioner in TIC to provide trauma-informed reproductive health services. While Owens et al. (2020) described that this training was on-the-job and mentorship- and discussion-based, the components of the said training were not clearly reported. Niebauer et al. (2021) stated that providers should consider emotionally therapeutic resources for breast cancer patients but did not describe how this could be achieved. Taking a social approach, one provider in Sayani et al.’s (2021) study provided TIC by placing living conditions and social needs at the center of patient care to enhance their social determinants of health and address the health impacts of structural violence, but it was unclear how they shifted the locus of care. Similarly, Suarez et al. (2021) described that TIC efforts should address structural discrimination to provide effective care through trauma screening and intervention efforts but did not provide examples of trauma screening tools or action items beyond referral. Overall, the literature was heterogeneous regarding applications of TIC across care settings and non-specific regarding actions taken to achieve implementation.

### Effectiveness and Feasibility of TIC

Five studies implemented TIC into practice, highlighting the impacts of incorporating trauma-informed medical practices into patient care (Andrejko & Katrichis, 2022; Garcia et al., 2017; Owens et al., 2020; Regal et al., 2020; Sayani et al., 2021). Three studies agreed that interprofessional collaboration in TIC improved understanding of patient’s physical and psychosocial needs (Andrejko & Katrichis, 2022; Garcia et al., 2017; Regal et al., 2020). In addition, an interprofessional care team was described to improve quality of life, maximize patient safety, and develop a sense of trust between patient and provider (Andrejko & Katrichis, 2022; Regal et al., 2020). The composition of interdisciplinary care teams varied, but it was suggested that nurses are particularly vital to recognize patient needs and facilitate a sense of safety and trust (Andrejko & Katrichis, 2022). Comparably, Regal et al. (2020) highlighted that the gender of providers can improve patient anxiety and pain management, specifically, when a female radiation oncologist and social worker were present during all cancer-related treatments. Furthermore, Owens et al. (2020) reported that women who received trauma-informed reproductive health services described feeling respected and listened to and reported overall positive experiences with clinicians. More broadly, Sayani et al. (2021) reported that the provision of TIC aided in empowering patients and managing health risks. The evidence was consistent in that the integration of TIC was beneficial for patients and their health.

As eight studies did not implement TIC into their practices, their descriptions of effectiveness and feasibility of TIC were either not reported (Suarez et al., 2021), or stated as an anticipated outcome (Currie et al., 2019; Dhawan & LeBlanc, 2022; Kazak et al., 2005, 2006; Kohler et al., 2021; Lawson & Lawson, 2018; Niebauer et al., 2021). Both Kazak et al. (2005, 2006) agreed that the use of TIC interventions should be brief, competency-based, non-stigmatizing, and aimed at the individual needs of the patients and their families. Furthermore, four studies agreed that the use of TIC could minimize the risk of re-traumatization occurring (Dhawan & LeBlanc, 2022; Kohler et al., 2021; Lawson & Lawson, 2018; Niebauer et al., 2021). Examples of avoiding re-traumatization included the creation of a safe environment and mitigation of racial disparities by hematologists (Dhawan & LeBlanc, 2022), the empowerment of patients during cervical cancer screening to have control to stop an exam (Kohler et al., 2021), and the alleviation of stress in breast cancer patients through the provision of psychosocial support (Niebauer et al., 2021). The utility of TIC was anticipated across forms of trauma, types of cancer, and care settings.

No study evaluated the feasibility of integrating TIC in the context of cancer. However, three studies agreed that clinicians possessed the skills to utilize trauma-informed toolkits without formal TIC training (Dhawan & LeBlanc, 2022; Kazak et al., 2005, 2006). This suggested that it may be relatively simple for clinicians to begin integrating TIC into their practice. However, further research is needed to determine true feasibility and to identify whether allied health professionals within interdisciplinary care team (e.g., nurses, pharmacists, social workers, etc.) benefit from toolkit implementation or require TIC-related training.

### Gaps in TIC

In total, eight studies included in the review did not address current gaps in knowledge of, applications of, or other neglected aspects of cancer-related TIC (Currie et al., 2019; Garcia et al., 2017; Kazak et al., 2005; Kohler et al., 2021; Niebauer et al., 2021; Owens et al., 2021; Sayani et al., 2021; Suarez et al., 2021). However, the remaining studies reported several gaps involving TIC. Kazak et al. (2006) noted a lack of conceptual models to guide care for traumatic medical experiences, although, it appears that some preliminary TIC theorizations have since evolved in response. While several authors agreed that an interprofessional team is essential for providing holistic TIC that addresses the complexities of patient needs (Andrejko & Katrichis, 2022; Garcia et al., 2017; Sayani et al., 2021), Regal et al. (2020) highlighted a lack of formal training in and guidelines for TIC in oncology settings. In addition, a lack of research investigating the efficacy of TIC within the context of cancer was noted (Dhawan & LeBlanc, 2022; Lawson & Lawson, 2018). Moreover, the linkages between trauma and overall outcomes remain understudied (Andrejko & Katrichis, 2022). Therefore, research is required to fill these knowledge gaps.

### Recommendations for Future Policy, Practice, and Research

Given extensive gaps in knowledge, all but two studies (Currie et al., 2019; Owens et al., 2020) provided recommendations for future practice and research regarding TIC in the context of cancer. No recommendations were issued in the context of policy.

#### In Practice

In practice, several authors recommended that an interprofessional team be deployed to address the psychosocial barriers that people living with cancer often experience (Andrejko & Katrichis, 2022; Kazak et al., 2005; Lawson & Lawson, 2018; Niebauer et al., 2021; Sayani et al., 2021). Specifically, several authors recommended that such care teams utilize a trauma-informed framework (Andrejko & Katrichis, 2022; Kazak et al., 2006; Kohler et al., 2021) to ensure that providers can efficiently recognize and respond to patient needs. To achieve this, it is integral that healthcare providers are sensitive to patient and family cancer treatment experiences due to the potential for trauma and re-traumatization (Kazak et al., 2005). Additionally, it was recommended that healthcare providers receive TIC training (Regal et al., 2020; Sayani et al., 2021) and learn how to screen for traumatic experiences (such as adverse childhood experiences) to successfully implement interventions tailored to individual needs (Regal et al., 2020; Suarez et al., 2021). Similarly, Kohler et al. (2021) stressed the importance of healthcare providers being confident in their skills to recognize signs of trauma to ensure appropriate care is provided to patients receiving cervical cancer screening, including referral to additional services. Additionally, Niebauer et al. (2021) recommended that healthcare providers deliver trauma-informed, stress-reducing support to individuals living with breast cancer to improve their psychological healing process. Furthermore, it was reported as integral that healthcare providers explore the impact of the disease at the family level in the context of pediatric cancer to deliver interventions aimed at enhancing resiliency within the family (Garcia et al., 2017).

#### In Research

With respect to research implications, several authors suggested new avenues be explored to better understand TIC in terms of its components, settings, and contexts (Dhawan & LeBlanc, 2022; Kazak et al., 2005; Lawson & Lawson, 2018; Niebauer et al., 2021). Additional research was deemed necessary to improve the individualized, person-centered, decision-making process of care (Dhawan & LeBlanc, 2022), further evaluate traumatic experiences to determine if a relationship exists between stress and breast cancer (Niebauer et al., 2021), and tailor multidisciplinary care to the needs of the patient (Kazak et al., 2005). Furthermore, additional research was considered warranted to evaluate the impact of trauma-informed screening for adverse childhood experiences in medical settings (Regal et al., 2020) and transgender and gender-diverse populations (Suarez et al., 2021). Additionally, further research was considered necessary to objectively determine which methods of TIC delivery and involvement of which trauma-informed care providers are most beneficial to promote the successful integration of TIC at individual, organizational, and system levels (Lawson & Lawson, 2018). It was also considered important that researchers explore the provider’s perception of patients’ experiences and outcomes after receiving TIC (Regal et al., 2020) to aid in the development of interventions across the care continuum. As an emerging area of research, there are opportunities to implement recommendations related to TIC and cancer.

## Discussion

This scoping review identified literature related to trauma-informed approaches to cancer in Canada and the United States populations. Eleven of thirteen articles identified were published within the past 5 years, which indicated that TIC in the context of cancer care is gaining interest. This growth was visible across cancer types and timing, as this review captured hematologic, cervical, breast, ovarian, and lung cancers at several points across the cancer-care continuum: cancer-risk behaviors, routine cancer screening, previous malignancy, and active malignancy. Only five articles operationalized TIC, of which all adopted SAMHSA’s (2014a) theorization in full (*n* = 3) or in part (*n* = 2). Across all studies, the majority (*n* = 11) issued recommendations for future TIC research and practice; however, the effectiveness of TIC in improving cancer outcomes and feasibility in providing TIC in cancer settings was not well-understood.

### Poor Consistency in Operationalization

One of the most important findings from this review is that most of the studies did not formally conceptualize or operationalize TIC. Only five studies included a definition on the concept of TIC and either directly adopted the definition as developed by SAMSHA (2014a) (Andrejko & Katrichis, 2022; Dhawan & LeBlanc, 2022; Lawson & Lawson, 2018) or adapted it to their needs (Regal et al., 2020; Sayani et al., 2021). While the SAMHSA definition is comprehensive, it was developed in the context of substance abuse and mental health care and resultantly, may lack important elements specific to the unique context of cancer-related trauma. The lack of consistency in conceptualization of TIC within the context of cancer lends concern to the integrity of the application and operationalization of TIC in this population. This was demonstrated by the trauma-informed interventional efforts proposed by Currie et al. (2019), as they appeared to be trauma-focused instead of trauma-informed. This was evidenced by the intervention directly addressing patients’ trauma instead of viewing trauma as a consideration in the context of a distinct issue. More broadly, a scoping review of T(V)IC by Wathen et al. (2021) noted that many authors note a lack of conceptual clarity regarding T(V)IC which presents a barrier to quality research. Based on these findings, it is imperative that future research focuses on developing an effective operationalization of trauma-informed approaches to cancer. A concept analysis would be particularly useful to determine the defining attributes of trauma-informed approaches to cancer care.

### Screening and Trauma-Informed Care

As is seen in the extant violence literature, this review lacked consensus regarding the ideal application of trauma-informed care and screening procedures, specifically, whether screening and TIC should be universal or targeted. Studies in this review advocated for universal trauma screening (Regal et al., 2020), targeted trauma screening (Kohler et al., 2021; Suarez et al., 2021), responsive TIC (Andrejko & Katrichis, 2022; Dhawan & LeBlanc, 2022), and universal TIC (Kazak et al., 2006; Suarez et al., 2021). The variation in recommendations is reflective of the broader literature, where screening recommendations vary by type of trauma measured, demographic characteristics, and type of health concern, with screening for gender-based violence and adverse childhood experiences as particular areas of debate (Raja et al., 2021). Moreover, it is yet to be determined whether inquiring about specific forms of trauma such as adverse childhood experiences or gender-based violence is conceptually aligned with TIC, given that it risks re-traumatization by asking patients to disclose specific traumas (Racine et al., 2020). A potentially more trauma-informed method of screening is proposed by Lewis-O’Connor et al. (2019), who advocated for a progressively narrow, “tiered” approach to trauma inquiry that does not require disclosure. A tiered approach to screening becomes specific only at the patient’s discretion. For example, inquiry may begin by asking, “Have you had any life experiences that you feel have impacted your health and well-being?” instead of inquiring about specific experiences of trauma, which grants the patient control over the potential invasiveness of the inquiry process. Alternatively, many scholars have argued that all care should be trauma-informed (Coles & Jones, 2009; Owens et al., 2021; Raja et al., 2021; Tillman, 2020), potentially eliminating the need for screening practices or trauma inquiry altogether. Prominent TIC theorizations proposed by Wathen and Varcoe (2021) and SAMHSA (2014a) also advocate for TIC as a universal approach that does not include trauma disclosure. Overall, however, further research is needed to identify what approach to screening and inquiry is preferred by patients (if any) and what approach to TIC provision is most useful and feasible for providers.

### Lack of Evidence-Based Recommendations

It is evident from this review that there are gaps in the literature regarding trauma-informed approaches to cancer in Canada and the United States. Despite this, many studies provided recommendations for the use of TIC in practice based on anticipated outcomes, despite a lack of evidence to support them. Anticipated impacts included a reduced risk of re-traumatization (Dhawan & LeBlanc, 2022; Kohler et al., 2021; Lawson & Lawson, 2018; Niebauer et al., 2021) and traumatic stress (Kazak et al., 2006), as well as optimized well-being (Garcia et al., 2017). These impacts are reasonable to predict; however, without the availability of randomized controlled trials or similarly rigorous evidence to evaluate efficacy and feasibility in desired populations and settings, stated impacts and subsequent recommendations should be interpreted with caution.

Additionally, it is important to discuss the conflicting opinions in the literature regarding the need for TIC training. Both Regal et al. (2020) and Sayani et al. (2021) agreed that healthcare providers should receive formal TIC training to understand how to effectively inquire about traumatic experiences and provide individualized, trauma-informed patient care. This aligns with broader trauma literature, which has underscored the need for clinical and non-clinical staff TIC training to help develop trust, build positive relationships, and increase treatment adherence (Menschner & Maul, 2016). However, within this review, several authors agreed that formal training is not necessary to provide effective TIC, as clinicians possess the skills to utilize trauma-informed toolkits within their practice settings (Dhawan & LeBlanc, 2022; Kazak et al., 2005, 2006). Overall, the literature is consistent in that providers can integrate TIC into their practice; however, the role of training in this transition remains contested. Future research should evaluate the role of training in the integration of TIC into practice across disciplines and providers.

### Future Research

This scoping review identified literature on trauma-informed approaches to cancer in Canada and the United States, in which a gap in knowledge regarding the conceptualization and application of trauma-informed approaches to cancer was obvious. Given this, there would be great benefit from randomized controlled trials and rigorous mixed-methods studies to evaluate the effectiveness of TIC in promoting patient outcomes (including mental and physical health outcomes/quality of life, treatment adherence, and survivorship care participation) across oncology settings. Moreover, feasibility studies should be completed to promote successful, sustainable clinician implementation and organizational support in the context of existing demands. In addition, studies that recommend TIC for practice and/or implement TIC should offer examples of clear, actionable items to facilitate a greater understanding of how TIC is applied in medical and community settings and provide a “toolbox” from which healthcare providers can draw upon to implement TIC as appropriate. Without compelling evidence regarding TIC efficacy and feasibility, and actionable items to facilitate application, care teams are unlikely to implement TIC. In the absence of cancer-specific TIC knowledge and guidance, academics and healthcare providers/organizations may consider consulting the broader TIC literature for information (e.g., Wathen and Varcoe (2021) and SAMHSA (2014a)). However, it would be ideal to have cancer-specific evidence to inform TIC decision-making in medical contexts. Therefore, it is integral that future research includes both patient and provider perspectives to ensure the successful development and integration of trauma-informed approaches to care.

Additionally, it is important to note that the high demand for TIC research in the context of research is global in scope, despite this review being limited to Canada and the United States. For example, Klepáčková and Černá (2020) recently advocated for a trauma-informed approach to palliative nursing care for oncology patients in the Czech Republic. In addition, Hughes et al. (2016) identified linkages between adverse childhood experiences and cancer in a sample of English and Welsh residents, which led them to recommend the development of trauma-informed services. Moreover, Sigurdardottir and Halldorsdottir (2018) identified a need for trauma-informed care training and education for clinicians in Iceland who work with patients who have experienced sexual abuse as a child. As the body of TIC literature in the context of cancer increases, a systematic review and meta-analysis of global research would be warranted to better understand TIC for cancer care around the world. A summary of recommendations for future research, practice, and policy is available in Supplemental Appendix B.

### Limitations

There are some limitations that should be considered alongside the findings of this review. First, the articles compiled were largely from the United States (US), as only three studies emerged from the Canadian context (Currie et al., 2019; Sayani et al. 2021; Suarez et al., 2021). Given diverse healthcare systems, medical curricula, and professional bodies of oncological care, it is unclear whether findings can be extrapolated from the US to the Canadian setting and vice versa. Overall, this review presents a stronger understanding of TIC in oncology in the US setting than its Canadian equivalent and geographical differences were not accounted for in our analysis. Second, it should be noted that the small number of articles compiled for this review may not be representative of the true state of TIC in oncology care. It is possible that TIC is being integrated into medical settings without also producing publications, or that TIC is being provided under a different term, such as “patient-centered care” or “equity-oriented care” without being explicitly linked to trauma. As such, conclusions about the popularity of trauma-informed cancer care in Canada and the US cannot be accurately drawn from this review alone. In addition, not all types of cancer were represented in this review, meaning that the merits of TIC across diverse cancer types are unknown. Some forms of cancer are considered more potentially traumatic than others due to threat to life (e.g., metastatic breast cancer vs. melanoma in situ), but this review did not differentiate between cancer types during analysis. Furthermore, the cancer continuum was not equally represented by the articles in this review, as the majority focused on active malignancy despite linkages between cancer and trauma in pre-malignancy, at diagnosis, and during recurrence, remission, and survivorship care. Consequently, this review is narrow in that few types of cancer and vantage points in the cancer continuum are represented—a limitation of the available research. Moreover, this review did not evaluate the quality of the articles because Arksey and O’Malley (2005) did not view quality appraisal as relevant to a scoping review. This review also did not evaluate the demographic diversity of the primary sources of evidence, so it is unclear if recommendations are generalizable. Given this, all evidence was treated as if it were of equal quality and merit, when this may not be the reality. As the literature continues to progress, a systematic review would be better suited to evaluating the diversity and quality of the data.

## Conclusion

Evidence regarding trauma-informed approaches to cancer in Canadian and US settings continues to evolve. While the literature largely agreed on the merits of a trauma-informed approach and recommended its integration across the cancer care continuum, even pre-diagnosis, the scarcity of literature suggested a lack of implementation. Further research is required to understand applications, effectiveness, and feasibility of TIC across cancer types, populations, the cancer continuum, and geographical settings. Given strong potential to improve cancer outcomes, it is pertinent that future research make TIC a priority.

## Supplemental Material

sj-docx-1-tva-10.1177_15248380221120836 – Supplemental material for Trauma-Informed Approaches in the Context of Cancer Care in Canada and the United States: A Scoping Review Click here for additional data file. Supplemental material, sj-docx-1-tva-10.1177_15248380221120836 for Trauma-Informed Approaches in the Context of Cancer Care in Canada and the United States: A Scoping Review by Cara A. Davidson, Kelly Kennedy and Kimberley T. Jackson in Trauma, Violence, & Abuse

sj-docx-2-tva-10.1177_15248380221120836 – Supplemental material for Trauma-Informed Approaches in the Context of Cancer Care in Canada and the United States: A Scoping Review Click here for additional data file. Supplemental material, sj-docx-2-tva-10.1177_15248380221120836 for Trauma-Informed Approaches in the Context of Cancer Care in Canada and the United States: A Scoping Review by Cara A. Davidson, Kelly Kennedy and Kimberley T. Jackson in Trauma, Violence, & Abuse
