# Supplementary material for: Trauma-Informed Approaches in the Context of Cancer Care in Canada and the United States: A Scoping Review
Source: Trauma Violence Abuse. 2022 Sep 9;24(5):2983–96. doi: 10.1177/15248380221120836 (PMC10594848; doi:10.1177/15248380221120836)
Supplement: sj-docx-1-tva-10.1177_15248380221120836 – Supplemental material for Trauma-Informed Approaches in the Context of Cancer Care in Canada and the United States: A Scoping Review [file sj-docx-1-tva-10.1177_15248380221120836.docx]

**Appendix A**

*Critical Findings*

| *Category* | *Findings* |
| --- | --- |
| Theorizations of TIC | - Trauma-informed approach definition by the Substance Abuse and Mental Health Services Administration (2014)   - Four assumptions of realizing the widespread impact of trauma and understanding potential paths for recovery; recognizing the signs and symptoms of trauma in clients, families, staff, and others involved with the systems; responding by fully integrating knowledge about trauma into practice, procedures, and practices; and resisting re-traumatization (Andrejko & Katrichis, 2022)   - Six principles of safety, trustworthiness and transparency, peer support, collaboration and mutuality, empowerment, voice, and change, and cultural, historical, and gender issues (Andrejko & Katrichis, 2022; Dhawan & LeBlanc, 2022, Lawson & Lawson, 2018) - Tertiary effort to prevent mental, social, and physical health consequences (Regal et al., 2020) - Equity-oriented care that acknowledges and addresses the health impacts of structural violence (Sayani et al., 2021) |
| Applications of TIC | - Trauma screening   - Universal screening (Regal et al., 2020)   - Targeted screening (Kohler et al., 2021; Suarez et al., 2021) - Trauma-informed care   - Universal TIC (Kazak et al., 2006; Suarez et al., 2021)   - Responsive TIC (Andrejko & Katrichis, 2022; Dhawan & LeBlanc, 2022 - Community-based interventions   - Trauma-informed yoga, drumming, and psychoeducation for at-risk adults (Currie et al., 2019)   - Pediatric medical traumatic stress model directed at families (Kazak et al., 2005, 2006)   - FOCUS theoretical model: an integrated approach to behavioural health screening and consultation for pediatric oncology families (Garcia et al., 2017) - Healthcare-based interventions   - Interprofessional care team ((Andrejko & Katrichis, 2022; Garcia et al., 2017; Kazak et al., 2006; Regal et al., 2020; Sayani et al., 2021)   - Hematologic care (Dhawan & LeBlanc, 2022)   - Cervical cancer screening (Kohler et al., 2021)   - Reproductive services (Owens et al., 2020)   - Breast cancer care (Niebauer et al., 2021)   - Social determinants of health (Sayani et al., 2021; Suarez et al., 2021) |
| Effectiveness and Feasibility of TIC | - Known outcomes   - Improved understanding of patient physical and psychosocial needs (Andrejko & Katrichis, 2022; Garcia et al., 2017; Regal et al., 2020)   - Interprofessional care team improved quality of life, patient safety, and sense of trust between patient and provider (Andrejko & Katrichis, 2022; Regal et al., 2020)   - Positive experiences with clinicians (Owens et al., 2020)   - Empowerment of patients and management of health risks (Sayani et al., 2021) - Anticipated outcomes   - Non-stigmatizing and individually tailored (Kazak et al., 2005, 2006)   - Minimize risk of re-traumatization (Dhawan & LeBlanc, 2022; Kohler et al., 2021; Lawson & Lawson, 2018; Niebauer et al., 2021) - No feasibility evaluations were reported |
| Gaps in TIC | - Lack of conceptual models (Kazak et al., 2006) - Lack of formal training in and guidelines for TIC in oncology (Dhawan & LeBlanc, 2022; Lawson & Lawson, 2018) - Linkages between trauma and outcomes not well-understood (Andrejko & Katrichis, 2022) |
| Recommend-ations for Future Policy, Practice, and Research | - In practice   - Interprofessional team to provide care (Andrejko & Katrichis, 2022; Kazak et al., 2005; Lawson & Lawson, 2018; Niebauer et al., 2021; Sayani et al., 2021).   - Use of trauma-informed framework for care (Andrejko & Katrichis, 2022; Kazak et al., 2006; Kohler et al., 2021; Niebauer et al., 2021)   - Sensitivity to patient and family cancer treatment experiences (Garcia et al., 2017; Kazak et al., 2005)   - TIC training for providers (Regal et al., 2020; Sayani et al., 2021)   - Screen for traumatic experiences (Regal et al., 2020; Sayani et al., 2021)   - Provider confidence in trauma recognition (Kohler et al., 2021) - In research   - Improve individualized decision-making process of care (Dhawan & LeBlanc, 2022)   - Evaluate whether a relationship exists between trauma and breast cancer (Niebauer et al., 2021)   - Tailor multidisciplinary care to patient needs (Kazak et al., 2005)   - Evaluate impact of trauma-informed screening for adverse childhood experiences (Regal et al., 2020) and transgender and gender diverse populations (Suarez et al., 2021)   - Determine which trauma-informed providers and methods are most successful to promote TIC at individual, organizational, and system levels (Lawson & Lawson, 2018)   - Explore the provider perception of patient experiences and outcomes after TIC (Regal et al., 2020) - In policy   - None reported |
