# Supplementary material for: Trauma-Informed Approaches in the Context of Cancer Care in Canada and the United States: A Scoping Review
Source: Trauma Violence Abuse. 2022 Sep 9;24(5):2983–96. doi: 10.1177/15248380221120836 (PMC10594848; doi:10.1177/15248380221120836)
Supplement: sj-docx-2-tva-10.1177_15248380221120836 – Supplemental material for Trauma-Informed Approaches in the Context of Cancer Care in Canada and the United States: A Scoping Review [file sj-docx-2-tva-10.1177_15248380221120836.docx]

**Appendix B**

*Implications for Practice, Policy, and Research*

| Practice | Policy | Research |
| --- | --- | --- |
| - Interprofessional care teams can improve TIC provision - Trauma-informed frameworks, toolkits, and/or provider training may improve responsiveness to patient needs and resist re-traumatization - Pediatric settings can consider a familial approach to TIC - TIC is potentially relevant throughout cancer (screening, diagnosis, treatment, remission) | - This review is not positioned to offer policy recommendations because no studies addressed policy | - Randomized controlled trials to evaluate the effectiveness of TIC in promoting patient outcomes - Feasibility studies from provider and organizational perspectives - Provision of actionable items for TIC implementation in community and medical settings - Systematic review and meta-analysis of global TIC cancer-related literature - Evaluate the role of training in TIC integration - Identify the preferred approach to screening (if any) by cancer patients - Concept analysis to determine the defining attributes of TIC in the context of cancer |
|  |  |  |
